# Glutamate receptor δ2 serum antibodies in pediatric opsoclonus myoclonus ataxia syndrome

**DOI:** 10.1212/WNL.0000000000006035

**Published:** 2018-08-21

**Authors:** Georgina Berridge, David A. Menassa, Teresa Moloney, Patrick J. Waters, Imogen Welding, Selina Thomsen, Sameer Zuberi, Roman Fischer, A. Radu Aricescu, Michael Pike, Russell C. Dale, Benedikt Kessler, Angela Vincent, Ming Lim, Sarosh R. Irani, Bethan Lang

**Affiliations:** From the Oxford Autoimmune Neurology Group (G.B., D.A.M., T.M., P.J.W., I.W., S.T., M.P., A.V., S.R.I., B.L.), Nuffield Department of Clinical Neurosciences, John Radcliffe Hospital, Oxford; Target Discovery Institute (G.B., R.F., B.K.), NDM Research Building, University of Oxford, Old Road Campus, Oxford; Paediatric Neurosciences Research Group (S.Z.), School of Medicine, University of Glasgow; Division of Structural Biology (A.R.A.), Nuffield Department of Clinical Medicine, University of Oxford, UK; Clinical Neuroimmunology (R.C.D.), Institute for Neuroscience and Muscle Research, University of Sydney, Australia; Children's Neuroscience Centre (M.L.), Evelina London Children's Hospital at St Thomas' NHS Foundation Trust, King's Health Partners Academic Health Science Centre, London; and Faculty of Medicine and Life Sciences (M.L.), King’s College London, UK.

## Abstract

**Objective:**

To identify neuronal surface antibodies in opsoclonus myoclonus ataxia syndrome (OMAS) using contemporary antigen discovery methodology.

**Methods:**

OMAS patient serum immunoglobulin G immunohistochemistry using age-equivalent rat cerebellar tissue was followed by immunoprecipitation, gel electrophoresis, and mass spectrometry. Data are available via ProteomeXchange (identifier PXD009578). This generated a list of potential neuronal surface cerebellar autoantigens. Live cell-based assays were used to confirm membrane-surface antigens and adsorb antigen-specific immunoglobulin Gs. The serologic results were compared to the clinical data.

**Results:**

Four of the 6 OMAS sera tested bound rat cerebellar sections. Two of these sera with similar immunoreactivities were used in immunoprecipitation experiments using cerebellum from postnatal rat pups (P18). Mass spectrometry identified 12 cell-surface proteins, of which glutamate receptor δ2 (GluD2), a predominately cerebellar-expressed protein, was found at a 3-fold-higher concentration than the other 11 proteins. Antibodies to GluD2 were identified in 14/16 (87%) OMAS samples, compared with 5/139 (5%) pediatric and 1/38 (2.6%) adult serum controls (*p* < 0.0001), and in 2/4 sera from patients with neuroblastoma without neurologic features. Adsorption of positive OMAS sera against GluD2-transfected cells substantially reduced but did not eliminate reactivity toward cerebellar sections.

**Conclusion:**

Autoantibodies to GluD2 are common in patients with OMAS, bind to surface determinants, and are potentially pathogenic.

Opsoclonus myoclonus ataxia syndrome (OMAS), also known as “dancing eye syndrome,” is a rare disorder that mainly affects children. OMAS is characterized by conjugate, asynchronous, multidirectional eye movements (opsoclonus), myoclonus, ataxia, behavioral and sleep disturbance, and sometimes cognitive decline.^1–3^ The clinical and imaging assessment of the disease suggest involvement of the cerebellum and/or pontine omnipause neurons. MRI in the acute phase is usually normal, but recently, patients with long-standing OMAS have been shown to have a reduction in the cerebellar gray matter volume, especially in the vermis and flocculonodular lobes, alongside a more generalized reduction in cortical thickness.^4^

In pediatric OMAS, the age at onset is typically within the relatively narrow 12- to 36-month age range.^2,5^ Furthermore, OMAS associates with an underlying neuroblastoma in approximately 50% of pediatric patients.^1,6^ Neuroblastoma is the most common solid tumor of childhood, derived from the sympathetic nervous system, and occurs almost exclusively in infancy and early childhood, with a median peak age between 18 and 24 months.^7^

While the precise pathogenesis of OMAS is undefined, the close association with neuroblastoma strongly suggests a paraneoplastic autoimmune process. B cell expansions with elevated levels of B cell activating factor have been shown in the CSF of patients with OMAS,^8,9^ and an HLA association has been established in some patients.^10^ Moreover, the neuroblastomas have marked lymphocytic infiltrates, akin to the thymic histology observed in early-onset myasthenia gravis.^11^ Finally, some studies describe binding of OMAS patient serum immunoglobulins to Purkinje cells, the surface of cerebellar dendritic arborizations, and to a few candidate neuronal proteins, although no reproducible antigenic targets have yet been established.^11–15^ Indeed, in one recent study, serum immunoglobulin G (IgG) precipitated 7 neuronal proteins found in neuroblastoma cell lines but none were shown to be direct targets of the autoantibodies.^16^

The striking overlap of symptom onset in OMAS and the peak age of neuroblastoma detection led us to hypothesize that this temporal juxtaposition was important in the pathogenesis of OMAS. Furthermore, the above observations strongly implicate cerebellar structures in disease etiology. Therefore, in our search for putative pathogenic autoantibodies in OMAS, we hypothesized an advantage to using cerebellar tissue representative of humans at approximately 2 years of age. Here, we combine immunohistology, immunoprecipitation, mass spectrometry, and bioinformatic techniques on age-equivalent rat cerebellar tissue and identified autoantibodies to the extracellular domain of glutamate receptor δ2 (GluD2) in the sera of pediatric patients with OMAS.

## Methods

### Patient material

OMAS serum samples (data available from Dryad, table 1, doi.org/10.5061/dryad.tq61224) were collected at diagnosis from 16 children (median age 2 years, range 1–8.5 years); further samples were available at 48 weeks in 5 of these patients. Eight (53%) were male and 11 (73%) had an associated neuroblastoma. As outlined in the table, disease control sera were available from children with new-onset epilepsy (median age 2.2 years, range 0.5–3 years, n = 78), Rasmussen encephalitis (age 8.2 years, range 1–18 years, n = 23) and autoimmune and other forms of encephalitis (age 8.25 years, range 0.4–15 years, n = 38), and from healthy adult controls (n = 37). Resected neuroblastoma tissue from one patient (18-month-old female) was available for study. Sera from 4 patients with neuroblastoma but without neurologic dysfunction (absence or presence of neurologic syndrome is a recorded field) were obtained from the Children's Cancer and Leukaemia and Tissue Bank, Leicester Royal Infirmary.

**Table:**
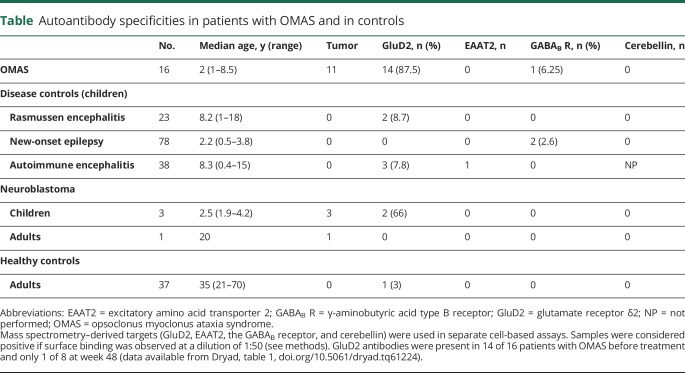
Autoantibody specificities in patients with OMAS and in controls

### Cerebellar tissue staining

Sprague-Dawley rats (P18, and adult) were perfused with saline (0.9%) under deep anesthesia. The brains were flash-frozen in isopentane at −40°C. Frozen rat brain sections (12 μm thick) were fixed with 4% paraformaldehyde (15 minutes at room temperature [RT]). Sections were incubated with sera or commercial antibodies (1:100–1:200) before incubation with biotinylated goat anti-human antibody (1:200; Vector Laboratories, Burlingame, CA). ABC complex (Elite kit; Vector Laboratories) and diaminobenzidine (0.5 mg/mL plus 0.03% of H_2_O_2_) were used to develop the reaction. For immunofluorescence, sections were incubated with commercial antibodies or serum (1:100–1:200) before fixation (15 minutes, RT). IgG binding was detected with a species-appropriate fluorescently labeled Alexa Fluor secondary antibody (ThermoFisher Scientific, Waltham, MA) and counterstained with DAPI (4',6-diamidino-2-phenylindole). Further details are presented in figure legends and data available from Dryad (Methods, doi.org/10.5061/dryad.tq61224).

### Neuroblastoma immunofluorescence

The neuroblastoma tissue from one patient (OMAS 15) was frozen in OCT (Fisher Healthcare). For immunofluorescence, neuroblastoma sections were incubated with commercial antibodies (1:200) and stained with a species-appropriate fluorescently labeled Alexa Fluor secondary antibody and counterstained with DAPI.

### Isolation of autoantigens

The 2 OMAS sera with the strongest binding to the cerebellum and the deep cerebellar nuclei (DCN) and pooled healthy control serum were used for the discovery of autoantigens via immunoprecipitation and analysis by mass spectrometry. Postnatal day 18 rat cerebellum was gently triturated and washed with phosphate-buffered saline, then incubated with 85 to 100 μL undiluted patient or control serum for 60 minutes with occasional inversion before the addition of solubilization buffer (150 mM NaCl, 10 mM Tris HCl, pH 7.4, 1% Triton X-100, protease inhibitor cocktail [P8340; Sigma-Aldrich, St. Louis, MO]) for 60 minutes on ice. The suspension was harvested after 2 rounds of centrifugation (2,000*g* for 5 minutes). Protein G Sepharose beads (Sigma) were added to the supernatant (3 hours at 4°C) to bind the bound antibody-antigen complexes, then extensively washed stepwise (150 mM through 1 M NaCl in solubilization buffer). The IgG-bound proteins were eluted by heating the Protein G Sepharose beads to 90°C in Laemmli sample buffer and the eluted proteins were electrophoresed (4%–12% sodium dodecyl sulfate gradient gel [WG1402; Invitrogen, Carlsbad, CA]). The protein bands were visualized with Imperial blue stain (ThermoFisher).

### Analysis by mass spectrometry

**Figure 1:**
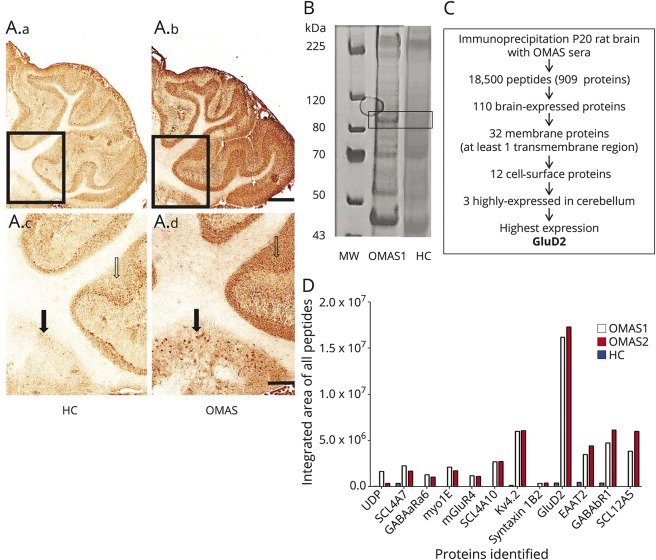
Autoantigen identification in OMAS (A) Serum immunoglobulin G (1:100) binding to rat cerebellar sections (12 μm) from HCs (A.a, A.c) or patients with OMAS (A.b, A.d). Strong staining is observed with OMAS sera in the granular layer (open arrow) and also in areas of the deep cerebellar nuclei (boxed area in upper panels, filled arrow in lower panels). Scale bars: 500 μm (A.a, A.b) and 200 μm (A.c, A.d). (B) Gel electrophoresis of postnatal day 20 rat cerebellum tissue immunoprecipitate. Eluted samples after immunoprecipitation with OMAS1 sera and pooled HC sera were run on a 4% to 12% sodium dodecyl sulfate precast gradient gel (WG1402; Invitrogen). (A) Unique band, approximate molecular weight of 100 to 110 kDa was seen exclusively in OMAS samples. The boxed area was excised for mass spectrometry. (C) Flow diagram for filters in mass spectrometry experiments. The mass spectrometry proteomics data have been deposited to the ProteomeXchange Consortium via the PRIDE partner repository^31,32^ with the dataset identifier PXD009578. (D) Identification of GluD2 as a putative autoantigen target in OMAS. Relative amounts of 12 surface-expressed neuronal proteins immunoprecipitated by 2 different OMAS sera (red/white bars) and not by HC (blue bars). For full description of the identified proteins, see data available from Dryad (table 2, doi.org/10.5061/dryad.tq61224). GluD2 = glutamate receptor δ2; HC = healthy control; MW = molecular weight; OMAS = opsoclonus myoclonus ataxia syndrome.

Eluates from the immunoprecipitation were prepared for liquid chromatography–tandem mass spectrometry by pooling the Laemmli sample buffer eluted immunoprecipitate fractions followed by chloroform-methanol precipitation. Mass spectrometry was performed using data-dependent acquisition on a Thermo Q Exactive mass spectrometer (data available from Dryad, Methods, doi.org/10.5061/dryad.tq61224). Methods for filtering of protein hits are illustrated in figure 1 and data available from Dryad (table 2, doi.org/10.5061/dryad.tq61224).

### Cell-based assays

Complementary DNA encoding the full-length human GluD2 mature polypeptide (GenBank ID NM_001510; Asp24-Ile1007) was cloned into the pHLsec vector (PMID: 17001101), immediately downstream of the secretion signal sequence and an external hemagglutinin (HA) peptide (YPYDVPDYA), and was used in a live cell-based assay (CBA) to detect antibody binding. Culture and staining procedures for the live CBAs were performed and scored as previously described.^17,18^ Sera (from 1:50) or commercial antibodies (1:750) were applied to live transfected cells for 1 hour at RT followed by 4% paraformaldehyde fixation, washing, and incubation with unlabeled goat anti-human Fc-specific antibody (1:750, Fisher A31125), and finally a third antibody layer with Alexa Fluor 568 donkey anti-goat IgG (1:750, Fisher A11057). Binding of commercial antibodies was detected using the appropriate species-specific secondary antibody (Alexa Fluor 568 rabbit anti-mouse A-11061 and Alexa Fluor 568 goat anti-rabbit A-11011).

### Standard protocol approvals, registrations, and patient consents

Ethics for this study was covered by the research ethics committee (REC) (16/YH/0013) and London-Fulham (13/LO/0706 NRES), Children's Hospital at Westmead (12/SCHN/395 and 09/SCHN/56), and Glasgow ERUK study (REC reference: 13/WS/0299). All animal work was conducted according to British Home Office regulations and under license (Home Office: 4003581).

### Data availability

Supplementary data are available from Dryad, doi.org/10.5061/dryad.tq61224. The mass spectrometry proteomics data have been deposited to the ProteomeXchange Consortium via the PRIDE partner repository^19,20^ with the dataset identifier PXD009578.

## Results

### OMAS serum IgG binding and downstream proteomic analyses

Initially, adult rat brain sections were used to look for antibodies in OMAS sera. This revealed a distinct pattern of immunoreactivity in 4 of the 6 samples tested. This pattern was characterized by widespread IgG immunoreactivity of the cerebellar cortex, especially within the granular layer, and strong IgG binding to the paravermal zone, where the DCN are located; the plane of the section includes the interposed nucleus. There was no evident staining in the white matter of the cerebellum (figure 1A). The 2 OMAS sera with the largest volume of serum available, which showed this pattern (5-year-old female and 2-year-old male; both with neuroblastoma), were used in the antigen discovery experiments.

Our previous attempts to identify putative antigens using tissue derived from embryonic or postnatal rat tissue (<P6) had proven unsuccessful (data not shown). Therefore, cerebellar tissue of postnatal rat pups (P17-20), considered to be age-equivalent to 18- to 24-month-old humans,^21^ were used instead. Precipitated OMAS IgG-antigen complexes were subjected to sodium dodecyl sulfate–polyacrylamide gel electrophoresis analysis and compared to a control sera (n = 5). A band of approximately 100 to 110 kDa was identified from OMAS patient gels (figure 1B). This region was excised from the OMAS and control gels and subjected to analysis by tandem mass spectrometry. The excised bands identified GluD2 in the patient but not control samples. The eluates typically contained approximately 18,000 peptides matching to several hundred proteins were isolated from gel bands. To identify targets of potentially pathogenic autoantibodies, stringent filters were applied to filter out proteins present in the controls, and from the unique ones, to identify cerebellar-specific membrane proteins with an extracellular domain. This reduced the putative target pool to 12 proteins (figure 1, C and D; and data available from Dryad, table 2, doi.org/10.5061/dryad.tq61224). Of these 12 proteins, GluD2, which shows high cerebellar/Purkinje cell specificity,^22^ was detected at approximately 3-fold-higher levels than any of the others proteins and was enriched by 20-fold as compared to healthy control samples.

### GluD2-specific autoantibodies

**Figure 2:**
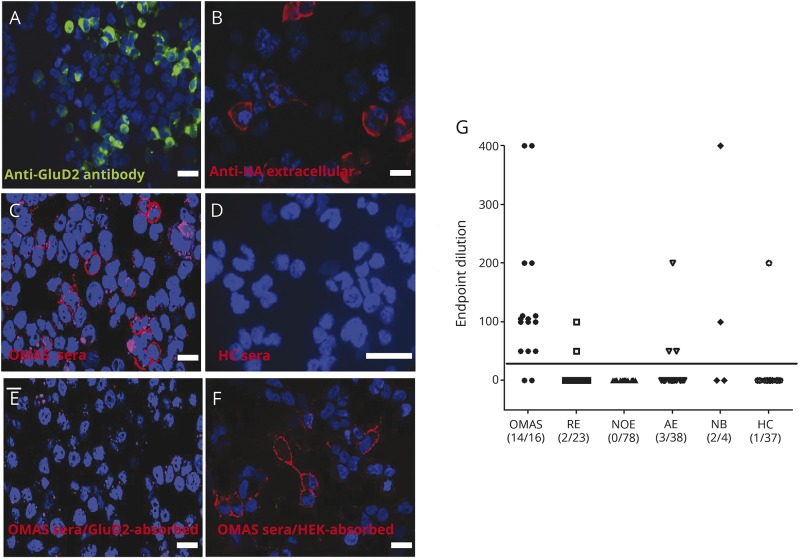
GluD2 live cell-based assay HEK 293T cells were transfected with complementary DNA encoding full-length GluD2, which had an extracellular HA tag. (A) Commercial antibody to GluD2 (1:200, D13266; Frontier Institute Japan, directed against an intracellular epitope) binds to permeabilized GluD2-transfected cells and surface expression of GluD2 tagged with HA was confirmed using an anti-HA antibody (B). Sera (1:50 dilution) from patients with OMAS (C) but not HCs (D) bound the surface of the GluD2-transfected cells. GluD2-reactive immunoglobulin Gs were removed after adsorption against GluD2-transfected (E) but not when adsorbed against mock-transfected (F) HEK cells. Scale bar = 10 μm. Graph (G) showing endpoint titration of all samples. Samples considered positive (solid line) if signal is observed at a titration of 1:40 or above.^17^ There is a significant difference between the groups (*p* < 0.0001; Kruskal-Wallis test). AE = autoimmune encephalitis; GluD2 = glutamate receptor δ2; HA = hemagglutinin; HC = healthy control; NB = neuroblastoma; NOE = new-onset epilepsy; OMAS = opsoclonus myoclonus ataxia syndrome; RE = Rasmussen encephalitis.

This identification of GluD2 as an autoantigen was confirmed using a CBA.^17^ HEK293T cells were transfected with complementary DNA encoding GluD2 fused to an extracellular HA tag. Expression of GluD2 was verified with a commercial antibody against the intracellular C-terminus of GluD2 (figure 2A; permeabilized CBA) and with a commercial antibody to the extracellular HA tag on the surface of live (nonpermeabilized) GluD2-transfected cells (figure 2B). Having established surface expression of GluD2, we observed that the OMAS sera (1:50 dilution) used in the antigen-discovery program, but not healthy control sera, contained IgG that bound GluD2-transfected live HEK cells (figure 2, C and D).

Overall, 14 of 16 (87.5%) OMAS samples bound the extracellular domain of GluD2-transfected cells with endpoint titrations between 1:50 and 1:400 (figure 2E). The 2 seronegative patients were male (11 and 18 months old), both with an associated neuroblastoma. In addition, 2 of the 4 (50%) sera from patients with neuroblastoma without neurologic features showed GluD2 antibodies (table). By contrast, in the control groups, only 5 of 139 (3.6%) of the pediatric neurologic controls had GluD2 antibodies (Rasmussen encephalitis 2/23, new-onset epilepsy 0/78, autoimmune encephalitis 3/38; *p* < 0.0001, Fisher exact test) (data available from Dryad, table 3, doi.org/10.5061/dryad.tq61224). One of the healthy controls showed binding (table).

To confirm antigenic specificity, GluD2-reactive OMAS sera were adsorbed either against GluD2-transfected or untransfected HEK cells. Only GluD2 adsorption eliminated the binding (figure 2, E and F). Furthermore, all OMAS sera were negative for IgG binding to EAAT2 (excitatory amino acid transporter 2), and cerebellin, identified at lower levels by the mass spectrometry (table; data available from Dryad, table 2, doi.org/10.5061/dryad.tq61224). However, γ-aminobutyric acid type B (GABA_B_)-receptor antibodies were detected in 1 of 15 OMAS and 2 of 139 disease controls. Samples at 48-week follow-up were available post immunotherapy from 8 patients with OMAS, 7 of which had been GluD2 antibody–positive at presentation. Only one sample remained GluD2 antibody–positive at 48 weeks in an asymptomatic patient (data available from Dryad, table 1, doi.org/10.5061/dryad.tq61224).

### GluD2 expression in cerebellum and neuroblastoma tissue

**Figure 3:**
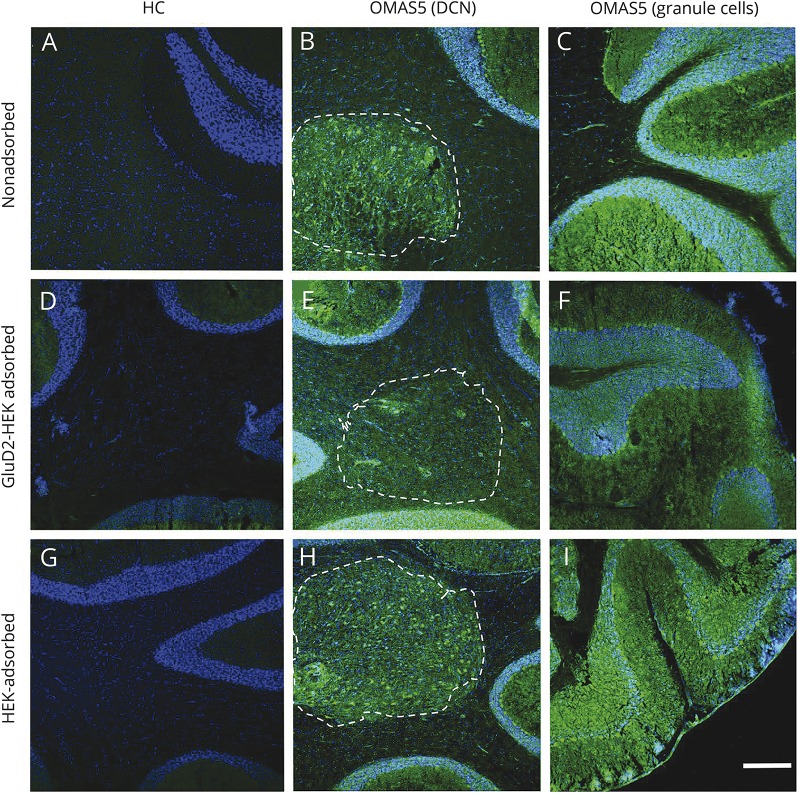
Binding of OMAS sera to GluD2 and other targets in cerebellar tissue Binding patterns of HC and patient with OMAS. OMAS sera (green) bound the DCN area (B) and granular cells (C) this binding was partially reduced after serum absorption against GluD2-transfected HEK cells (E and F) and unchanged after adsorption against untransfected HEK cells (H and I). The plane of the section that has been consistently cut usually included the interposed nucleus of the DCN area outlined by the dashed line; as no staining was seen in sections A, D, or G, no line was drawn. Similar results are shown for the bind serum dilution 1:100. Antibody binding was visualized by Alexa Fluor 488 goat anti-human (1:750, Fisher A-11013) and counterstained with DAPI. Scale bar = 100 μm. DAPI = 4',6-diamidino-2-phenylindole; DCN = deep cerebellar nuclei; GluD2 = glutamate receptor δ2; HC = healthy control; OMAS = opsoclonus myoclonus ataxia syndrome.

**Figure 4:**
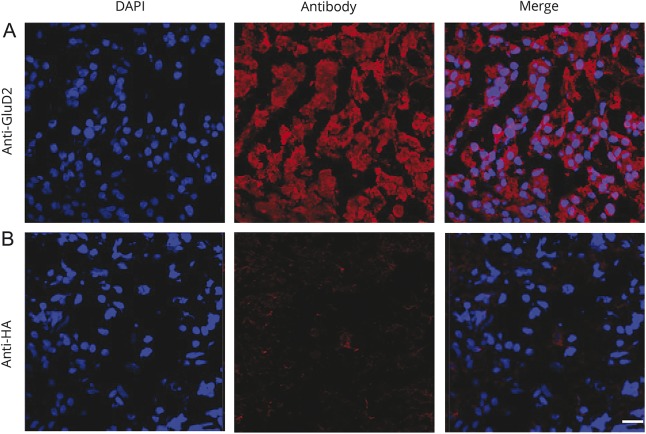
Expression of GluD2 in OMAS neuroblastoma tissue Sections of neuroblastoma from a child with OMAS who had serum GluD2 antibodies were incubated in commercial antibodies (A) (rabbit anti-GluD2, D13266; Frontier Institute Japan) or mouse anti-HA (B) (H3663, Sigma-Aldrich) at a dilution of 1:200. The sections were fixed (15 minutes in 3% PFA) and stained with a species-appropriate secondary antibody (Alexa Fluor 568 rabbit anti-mouse A-11061; or Alexa Fluor 568 goat anti-rabbit A-11011) and counterstained with DAPI. The sections demonstrate the presence of GluD2 in the tumor. Scale bar: 25 μm. DAPI = 4',6-diamidino-2-phenylindole; GluD2 = glutamate receptor δ2; HA = hemagglutinin; OMAS = opsoclonus myoclonus ataxia syndrome; PFA = paraformaldehyde.

In light of these findings, we revisited the cerebellar staining (figure 3). Application of GluD2-adsorbed sera to rat cerebellar sections revealed a marked reduction of staining in the granular area and at the site of the interposed nucleus of the DCN. However, residual staining was still observed in the 2 sera in which sufficient quantities remained for further testing (OMAS 5, OMAS 14). Also, a neuroblastoma from one of the GluD2-positive OMAS patients (OMAS 15) bound the commercial anti-GluD2 antibody indicating the presence of GluD2 within that tumor (figure 4).

Taken together, these results indicate that autoantibodies to GluD2 are frequently present in OMAS sera and target interposed nuclei of the DCN and other cerebellar structures. However, residual staining after GluD2-specific adsorption implies the presence of additional, as yet unidentified, antibodies that target similar brain regions.

## Discussion

Several convergent datasets strongly suggest OMAS has an autoimmune basis.^1,8,9,12–16^ However, despite several efforts to date, target antigens have remained elusive. In this study, mass spectrometry and bioinformatic techniques using age-equivalent cerebellar tissue were used to identify GluD2 as an autoantigen in OMAS. The expression of GluD2 in neuroblastoma tissue taken from a GluD2 sera–positive patient with OMAS was confirmed by immunofluorescence. Given data implicating cerebellar structures in disease pathogenesis, antigen-specific modulation of GluD2 may underlie some features of OMAS. The results support that antibodies that bind the extracellular domain of GluD2 may be a potentially pathogenic antibody in pediatric OMAS. However, the IgG cerebellar reactivities observed after GluD2-IgG absorption suggest it is not the sole potentially pathogenic agent in OMAS, and future studies should aim to define these other autoantigens. Nevertheless, links between GluD2 and several aspects of OMAS offer intriguing insights and are discussed in more detail below.

The ionotropic GluD2 is a cerebellar-specific receptor involved in synaptic organization and thus is an appropriate target for antibodies in OMAS. Children with mutations in the GluD2 gene (*GRID2*) show developmental delay, a loss of acquired motor skills, ocular apraxia, cerebellar ataxia, and cerebellar atrophy.^23,24^ GluD2 is highly expressed on the dendritic spines of Purkinje cells. These cells project GABAergic neurons into the vermis and DCN, the output cells of the cerebellum. Modulation of these projections may alter circuitry of the cerebellum (vermis and fastigial nuclei), the inferior olives, and the brainstem saccade premotor neurons (excitatory and inhibitory burst neurons, and omnipause neurons).^25^ Indeed, GluD2-deficient mice, with fewer functional synapses between the parallel fibers and Purkinje cells, have involuntary spontaneous eye and limb movements.^26^

GluD2 is especially highly expressed at the parallel fiber-Purkinje cell synapse. At this synapse, GluD2 interacts with cerebellin,^27^ a molecule that we also found in the immunoprecipitates from patient IgG-GluD2 complexes. Indeed, by linking GluD2, neuroblastomas, and the cerebellar nuclei, our data generate several hypotheses offering potential insights into OMAS etiology and pathogenicity. First, OMAS is a very rare condition and pediatric onset is most often within a very narrow temporal window of 12 to 48 months. It is known that in this early period, GluD2 expression rises in the cerebellum,^22^ and concurrently, neuroblastomas, which we show can express GluD2, are also maturing. It may be this ectopic expression in the neuroblastoma that breaks immunologic tolerance and leads to GluD2 autoantibodies, which can auto react with brain structures. Second, given the IgG staining pattern observed with the OMAS sera, the brain structures that would be targeted by the antibodies include focal cerebellar nuclei. These nuclei have roles in saccadic eye movements, omnipause neuron function, and ataxia.^25^ The origin of the myoclonus in OMAS is not well explained on the basis of a purely cerebellar dysfunction and this aspect requires further investigation.

GluD2 autoantibodies have been reported previously, although largely using methodology that favors detection of autoantibodies against intracellular epitopes. Several single or small case reports have described antibodies to GluD2, and other glutamate-receptor subtypes, mainly in adult patients with cerebellitis and encephalitis. ^28–30^ However, the peptide-based ELISAs used are unlikely to detect antibodies that react with the surface of native neuronal proteins. By contrast, in one patient with transverse myelitis following allogenic stem cell transplantation, patient serum IgG stained both the cerebellar molecular layer and GluD2-transfected HEK cells. In this study, GluD2 antibodies were not detected in approximately 300 disease and healthy controls.^31^

The selection of patient sera and the starting material were both critical in this study. The chosen sera bound to specific areas of the cerebellum, particularly the DCN, while the cerebellar tissue used for the mass spectrometry experiment was obtained from rats at an age equivalent to 18 to 24 human months. Previous experiments using fetal material had been unsuccessful, which is consistent with the very low expression of GluD2 before birth, in both rodents and humans, and its rapid increase post partum.

Despite being the first autoantigen with pathogenic potential described at high frequency in a substantial cohort of patients with OMAS, our study has several limitations. First, albeit only studied in a subset of patients, the antibody frequently disappeared rapidly following immunotherapy. However, we are aware of one patient in whom it persisted for >18 years of active disease.^32^ The small sample size (n = 8) and later serial sampling (48 weeks from disease onset) did not permit evaluation of correlation of antibodies to disease activity. Second, the serum GluD2 antibody levels were not very high (maximum 1:400), although, in general, this can depend on the relative and native cell-surface expression of antigenic proteins. In addition, this study only examined serum. CSF may have offered additional information. Third, GluD2 antibodies were not unique to children with OMAS but were also found in 2 of 4 children with neuroblastoma but without neurologic disease. We speculate that these antibodies, which may have increased in response to the underlying tumor are nonpathogenic, not at a critical threshold for that individual, or unable to gain antigenic access through the blood-brain barrier. Nevertheless, this may be biologically plausible as the antibody may have been induced by the presence of an underlying tumor. The antibody was also found in a small number (3.6%) of neurologic controls; the tumor status in all but one of these patients was unknown. However, the significantly increased occurrence of these antibodies vs controls (87.5% vs 3.6%) offers a potentially useful diagnostic test in OMAS. Finally, despite a marked reduction in cerebellar staining after GluD2-reactive IgG removal, some staining remained when reapplied to cerebellar sections, indicating the presence of further autoantibodies to additional antigenic targets.

Taken together, our findings provide possible mechanistic explanations for the site of the lesion in OMAS, the characteristic age at OMAS onset, and the relationship between the tumor and the immune system. Antibodies to surface-expressed GluD2 could identify a therapy-responsive disorder that would benefit from early treatment and tumor surveillance.

## Glossary

CBA: cell-based assay
DAPI: 4',6-diamidino-2-phenylindole
DCN: deep cerebellar nuclei
GABA: γ-aminobutyric acid
GluD2: glutamate receptor δ2
HA: hemagglutinin
IgG: immunoglobulin G
OMAS: opsoclonus myoclonus ataxia syndrome
RT: room temperature
